# Elephantiasis in pregnancy

**DOI:** 10.11604/pamj.2019.33.182.19564

**Published:** 2019-07-09

**Authors:** Ioannis Tsakiridis, Apostolos Mamopoulos

**Affiliations:** 1 Third Department of Obstetrics and Gynaecology, Faculty of Medicine, Aristotle University of Thessaloniki, Thessaloniki, Greece

**Keywords:** Elephantiasis, pregnancy, edema

## Image in medicine

A 33-year-old primiparous woman was admitted for induction of labor at 39+2 gestational weeks due to gestational diabetes mellitus, treated with insulin. During physical examination, elephantiasis in both legs was revealed. Most common causes of this condition include infections and lymphadenectomy. In our case it first appeared at the age of seven following an ankle injury. Originally the edema affected only the right leg, but during pregnancy it also affected the left leg, with hypertrophy of the skin and subcutaneous tissues. There was excessive weight gain of 52kg during pregnancy (body mass index on admission: 49.6kg/m^2^), mainly due to the leg edema. Elephantiasis is neither an indication for intensive fetal monitoring during labor, nor for cesarean delivery. In our case, the woman delivered by emergency cesarean section, due to intrapartum fetal compromise, without any further complications. The edema remained almost unchanged at six weeks' postpartum evaluation.

**Figure 1:**
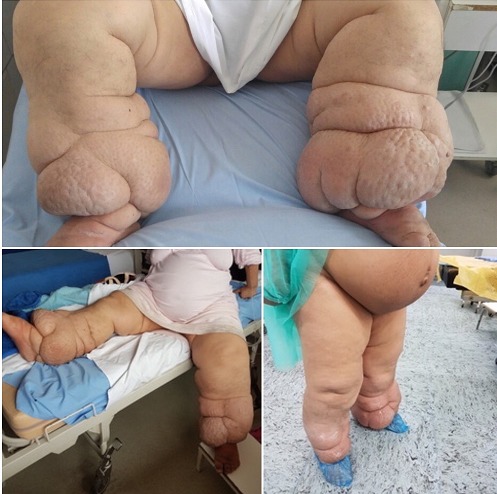
Elephantiasis in a pregnant woman with gestational diabetes mellitus

